# The Dynamic Recrystallization and α Texture Evolution of As-Sintered GNPs/TA15 Composites During Extrusion

**DOI:** 10.3390/ma18235398

**Published:** 2025-11-30

**Authors:** Zongan Li, Shuo Wu, Yongkang Fu, Jingxin Zhou, Liran Sun, Jiabin Hou, Chao Cui, Xiaocong Li, Zhikun Li

**Affiliations:** 1 School of Navigation and Shipping, Shandong Jiaotong University, Weihai 264209, China; 2 School of Materials Science and Engineering, and School of Ocean Engineering, Harbin Institute of Technology at Weihai, Weihai 264209, China; 3 Beijing Institute of Aerospace Systems Engineering, Beijing 100076, China

**Keywords:** GNPs, Ti matrix, dynamic recrystallization, texture, extrusion ratios

## Abstract

During the initial extrusion stage of the as-sintered GNPs/TA15 composite, dislocations accumulated along the GNPs and grain boundaries, leading to the formation of a subgrain structure. As extrusion progressed, these subgrains underwent rotation and transformed into finer grains through dynamic recrystallization. This process resulted in significant grain refinement, with the average grain size decreasing from 3.04 μm to 1.30 μm. Concurrently, the Ti matrix adjacent to the GNPs initially flowed towards the GNPs and subsequently elongated along the extrusion direction (ED). Furthermore, the deformed α grains experienced slip along the {10$\bar{1}$0}〈11$\bar{2}$0〉 system, giving rise to the development of [10$\bar{1}$1]//ED and [20$\bar{2}$1]//ED α textures. This study elucidates the influence of GNPs on the microstructural evolution, particularly in terms of grain refinement and the formation of α textures, as a function of increasing extrusion ratios.

## 1. Introduction

Graphene nanoplatelets (GNPs) exhibit remarkable advantages in mechanical properties and physical characteristics, including exceptional ultimate tensile strength, high specific surface area, and excellent flexibility [1]. Owing to these superior attributes, GNPs have been recognized as an ideal reinforcement for various metal matrices, such as Al [2], Cu [3], Mg [4], and Ti [5]. Both microstructure and GNPs play pivotal roles in determining the performance of composites, making it essential to investigate their evolution during processing. While the evolution of GNPs has been extensively studied in our previous work, microstructural evolution warrants further exploration [5]. Mu [6] examined the microstructural evolution of GNPs/Ti composites with increasing GNPs content, highlighting its critical influence on achieving superior mechanical properties. Their findings indicated that the obstruction of slip by GNPs led to the formation of {10$\bar{1}$0}〈10$\bar{1}\bar{2}$〉 compressive twinning in the Ti matrix. Additionally, high-density dislocations were observed to transform into a subgrain structure, accompanied by the development of low-angle grain boundaries during rolling. Cao [7] further demonstrated that rolling and annealing processes resulted in a significant reduction in average grain size from 15 μm to 5 μm, indicative of substantial grain refinement. Nevertheless, the underlying mechanisms of grain refinement and texture formation during deformation, particularly the role of GNPs, remain insufficiently explored and are crucial for a comprehensive understanding.

Zhang [8] successfully employed a transition-state billet to investigate the evolution of inhomogeneous reinforced structures in TiBw/Ti-6Al-4V composites, providing valuable insights. Their study revealed that during extrusion, TiB whiskers preferentially aligned and grew along the extrusion direction under high pressure and severe plastic deformation. This pioneering approach offers an effective methodology for elucidating the microstructural evolution of GNPs/Ti composites. Inspired by this work, we adopted a similar transition-state billet strategy to systematically investigate the influence of GNPs on microstructural evolution. The transition-state billet of GNPs/TA15 composite was fabricated by spark plasma sintering (SPS) at 900 °C for 10 min, followed by canned extrusion at 900 °C. This work establishes a fundamental foundation for understanding the deformation behavior of GNPs/Ti composites, guiding the optimization of their mechanical properties. Despite progress in understanding GNPs/Ti composites, the real-time influence of GNPs on microstructural evolution during deformation remains unclear, as studies primarily focus on initial and final states. Our work fills this gap by adopting a new transitional state. This approach uniquely enables a direct comparison within a single sample, elucidating how GNPs actively drive grain refinement and texture development under severe plastic deformation.

## 2. Experiment

**Figure 1:**
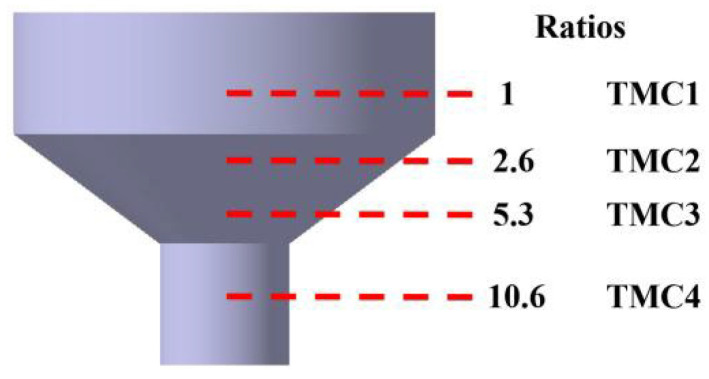
Transition-state billet of GNPs/TA15 composites.

The fabrication process of GNPs/TA15 composites involved three main stages: powder preparation, spark plasma sintering (SPS), and hot extrusion. Initially, TA15 (Ti-6.5Al-2Zr-1Mo-1V) powders with an average size of 120 µm were mixed with graphene nanoplates (GNPs) having a D50 of 6.423 µm and a thickness of 6 nm. In the first stage, 0.4 wt.% GNPs and TA15 powders were subjected to low-energy ball milling in absolute alcohol under an argon atmosphere. The mixed powders were homogenized using a mechanical agitator, and the resulting slurry was vacuum-filtered and dried. In the second stage, the dried powders were loaded into a graphite mold with dimensions of 40 mm (inner diameter) × 100 mm (height) and consolidated using SPS at 900 °C for 10 min under vacuum, resulting in an as-sintered billet. For the final stage, the as-sintered billet was encapsulated in a 45# steel can (outer diameter: 52 mm) and preheated at 900 °C for 30 min in a high-temperature box furnace. To investigate the deformation behavior, a transition-state billet with a continuous deformation gradient (from 52 mm to 16 mm in diameter) was prepared by controlling the half hydraulic stroke, as illustrated in Figure 1. (The extrusion process was carried out using a 315 T press at a speed of 0.5 m/s. The die, with a nominal angle of 70°, was maintained at 300 °C. Following extrusion, the sample was air-cooled at room temperature.) Additionally, extruded samples with different extrusion ratios of 1 (TMC1), 2.6 (TMC2), 5.3 (TMC3), and 10.6 (TMC4) were obtained for further analysis.

The phase composition of the composites, including α-Ti, β-Ti, and TiC, was characterized using X-ray diffraction (XRD, Rigaku Corporation, Tokyo, Japan). The structural integrity and phase characteristics of GNPs were further verified by Raman spectroscopy (Renishaw plc, Wotton-under-Edge, UK) with a 532 nm laser excitation source. Microstructural analysis was performed using scanning electron microscopy (SEM, MERLIN, Carl Zeiss, Oberkochen, Germany) equipped with an electron backscattered diffraction (EBSD) system to investigate grain orientation and texture evolution. The XRD data were Rietveld refined by the least squares method using GSAS-II (version 2.1). Additionally, the mechanical properties were evaluated through microhardness testing using a Vickers indenter (MHVS-1000Z, SuLiang Instrument Technology (Suzhou) Co., Ltd., Suzhou, China), providing insights into the local deformation behavior of the composites.

## 3. Results and Discussion

**Figure 2:**
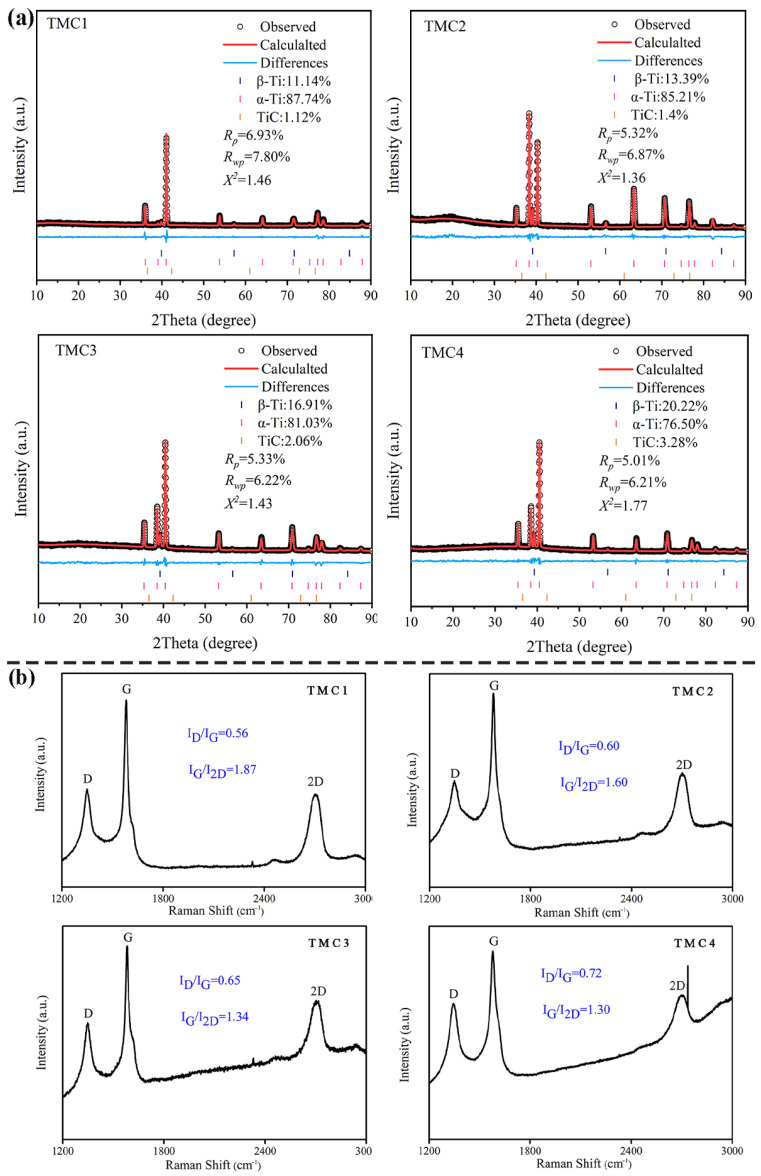
Rietveld analysis of the XRD patterns of TMCs (**a**); Raman spectra patterns of TMCs (**b**).

Figure 2 presents the Rietveld analysis of the XRD patterns and Raman spectra of the TMCs. As shown in Figure 2a, the diffraction peaks correspond to the α-Ti, β-Ti and TiC phases, while no signals of GNPs or other in situ phases are detected. With the increase in the extrusion ratio, the content of α-Ti gradually decreases, while the contents of β-Ti and TiC gradually increase. To further characterize the structural features of GNPs, Raman spectroscopy was employed, as illustrated in Figure 2b. The spectra revealed distinct D, G, and 2D bands, where the intensity ratio ID/IG reflects the defect density, and IG/I2D correlates with the number of graphene layers [9]. With increasing extrusion ratios, the ID/IG ratio exhibited a gradual increase from 0.56 to 0.72, while the IG/I2D ratio decreased progressively from 1.87 (indicating more than 5 layers) to 1.30 (indicating fewer than 5 layers). These trends suggest that the extrusion process reduced the number of GNPs layers and increased structural defects [10], a phenomenon attributed to interlayer slipping and delamination of GNPs during deformation. Consistent with this observation, Hou [5] reported that interlayer slipping promotes in situ reactions between GNPs and the Ti matrix, leading to an increase in defect density (ID/IG). Simultaneously, the slipping process reduces the thickness of GNPs by delamination.

**Figure 3:**
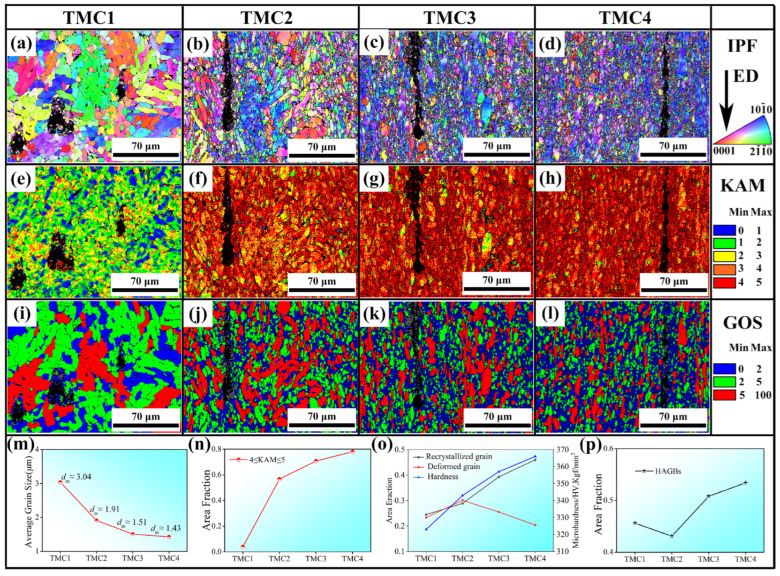
The IPF mappings of TMCs (**a**–**d**); KAM mappings (**e**–**h**); GOS mappings (**i**–**l**); Average grain size chart (**m**); KAM value between 4 and 5 chart (**n**); Recrystallized and deformed grains and microhardness chart (**o**); High-Angle grain boundary (HAGBs) chart (**p**).

Figure 3a–d displays the inverse pole figure (IPF) maps of α-Ti along the extrusion direction (ED), revealing two distinct types of grains: elongated grains and equiaxed grains. As shown in Figure 3a, fine grains are predominantly distributed near GNPs, while coarse grains are located farther away. During reheating, grain growth occurs through grain boundary migration; however, GNPs act as effective barriers to this migration, thereby inhibiting grain growth. Figure 3b–d illustrate that a significant number of equiaxed grains surround GNPs and elongated grains. The statistical analysis in Figure 3m demonstrates a clear trend of grain size reduction, with the average grain size decreasing from 3.04 μm to 1.30 μm, indicative of grain refinement. This phenomenon is likely associated with dynamic recrystallization [11], further enhanced by the Zener pinning effect exerted by the finely dispersed TiC particles, which effectively inhibit grain boundary migration and stabilize the refined microstructure. To substantiate this argument, kernel average misorientation (KAM) and grain orientation spread (GOS) maps were analyzed. Figure 3e–h present KAM maps with color-coded misorientation angles, where regions with KAM values between 4 and 5 (highlighted in red) represent areas of high dislocation density. The corresponding chart in Figure 3n shows a pronounced increase in dislocation density with higher extrusion ratios (The dislocation density of TMC1 is 2.9 × 10^14^ m^−2^, that of TMC2 is 4.9 × 10^14^ m^−2^, that of TMC3 is 5.1 × 10^14^ m^−2^, and that of TMC4 is 5.3 × 10^14^ m^−2^.). This significant dislocation accumulation is primarily driven by the strain incompatibility between the GNPs and the deformable Ti matrix. During extrusion, GNPs act as non-deformable obstacles, effectively blocking dislocation glide and promoting multiplication and pile-up around them. Notably, as seen in Figure 3f, high-density dislocations preferentially accumulate at grain boundaries and in regions adjacent to GNPs [10]. This process is concurrently influenced by the local strain incompatibility introduced by the TiC phases, which promotes heterogeneous deformation and contributes to the complexity of the overall texture evolution. During extrusion, dislocations multiply and migrate, but their movement is impeded by GNPs and grain boundaries, leading to dislocation pile-up and the formation of high-density dislocation regions. The presence of rigid TiC particles introduces additional barriers to dislocation motion, further amplifying the accumulation of dislocations and the formation of substructures.

Additionally, GOS maps in Figure 3i–l were used to distinguish recrystallized grains (GOS ≤ 2, highlighted in blue) from deformed grains (GOS ≥ 5, highlighted in red) [12]. Recrystallized grains are predominantly located near GNPs and deformed grains, and their fraction increases significantly with extrusion (the recrystallization ratio of TMC1 is 25%, that of TMC2 is 29%, that of TMC3 is 39%, and that of TMC4 is 46%.), as quantified in Figure 3o. This spatial correlation highlights the critical role of GNPs in promoting recrystallization through the particle-stimulated nucleation (PSN) mechanism. The high-strain zones surrounding the GNPs provide the necessary energy for the nucleation of new, strain-free grains. This further supports the occurrence of dynamic recrystallization, which contributes to grain refinement. Moreover, Figure 3p illustrates the evolution of high-angle grain boundaries (HAGBs), showing an initial decline followed by a rapid increase in HAGB fraction. Hou [13] attributed this behavior to the formation of low-angle grain boundaries (LAGBs) due to dislocation pile-up during deformation, which subsequently transform into HAGBs through dynamic recrystallization. In the initial stages of extrusion (e.g., TMC2), dislocations accumulate near GNPs and grain boundaries, forming dislocation substructures. With continued extrusion (e.g., TMC3 and TMC4), these substructures evolve into subgrains, and LAGBs gradually transform into HAGBs, a process characteristic of dynamic recrystallization.

**Figure 4:**
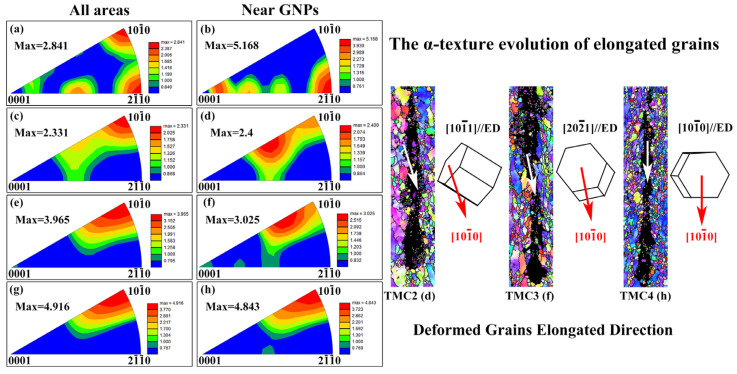
The α-Ti IPF in ED of all areas and the area near GNPs: (**a**,**b**) TMC1, (**c**,**d**) TMC2, (**e**,**f**) TMC3, (**g**,**h**) TMC4. And the area near GNP corresponding crystal orientation.

Figure 4 presents the inverse pole figure (IPF) maps of α-Ti along the extrusion direction (ED) for TMCs, covering both the overall regions and the areas adjacent to GNPs. As shown in Figure 4a, the [10$\bar{1}$0]//ED and [2$\bar{1}\bar{1}$0]//ED α textures are clearly observed. For TMC1, which underwent no deformation, the α texture is solely influenced by the β→α phase transformation, adhering to the Burgers orientation relationship of {0001}//{110} and 〈111〉//〈11$\bar{2}$0〉 [14]. However, only a strong [2$\bar{1}\bar{1}$0]//ED α texture is evident in Figure 4b. This can be attributed to the significantly larger average grain size of TMC1 compared to other samples (Figure 3a), resulting in fewer grains distributed around GNPs. Consequently, the α texture in Figure 4b, mapped from a limited number of grains, exhibits a pronounced intensity. Furthermore, Figure 4c,e,g,h reveal a consistent [10$\bar{1}$0]//ED α texture, with the maximum texture intensity increasing from 2.331 to 3.965 and then to 4.916 as the extrusion ratio rises. The presence of GNPs significantly influences this texture evolution by introducing heterogeneous deformation. The constrained deformation around the rigid particles leads to complex local crystal rotations, which stabilizes the [1010]//ED orientation and contributes to the overall texture strengthening. This trend can be explained by three factors: (I) During air cooling, the transformation of β grains into α grains follows the Burgers relationship, leading to the formation of a [10$\bar{1}$0]//ED α texture. (II) Under extrusion forces, the primary α grains undergo deformation primarily through prismatic glide ({10$\bar{1}$0}〈11$\bar{2}$0〉), which contributes to the development of the [10$\bar{1}$0]//ED α texture [15]. (III) Recrystallized grains inherit the texture of deformed grains due to the oriented nucleation mechanism. Notably, the fraction of recrystallized α grains increases significantly with higher extrusion ratios. Additionally, the IPF maps near GNPs for TMC2 and TMC3 (Figure 4d,f) exhibit distinct differences compared to the overall regions, with a [10$\bar{1}$1]//ED α texture observed in Figure 4d and a [20$\bar{2}$1]//ED α texture in Figure 4f.

As illustrated in Figure 4, the elongated direction of deformed grains gradually aligned with the extrusion direction (ED), eventually becoming parallel. The crystallographic orientation [10$\bar{1}$0] of TMCs is indicated by red arrows in the figure. Notably, the [10$\bar{1}$0] directions in both [10$\bar{1}$1]//ED and [20$\bar{2}$1]//ED crystal orientations are parallel to the elongated direction of the deformed grains. This alignment can be attributed to the dominant deformation mechanism of prismatic glide {10$\bar{1}$0}〈11$\bar{2}$0〉 in the deformed grains [15]. Therefore, the evolution of α texture is closely associated with the grain elongation direction, which reflects the flow direction of the Ti matrix near GNPs. Under the combined action of extrusion force and die constraints, the Ti matrix initially flows to fill voids and subsequently aligns with the ED. Specifically, the Ti matrix adjacent to GNPs first reorients towards the GNPs and then elongates along the ED. Concurrently, the α grains undergo slip along the {10$\bar{1}$0}〈11$\bar{2}$0〉 system, leading to the formation of [10$\bar{1}$1]//ED and [20$\bar{2}$1]//ED α textures with distinct elongated directions.

The microstructural evolution observed in this study, particularly the significant grain refinement, high-density dislocation structures, and the introduction of abundant GNPs matrix interfaces, not only strengthens the composite but also suggests promising functional potential. These refined microstructural features are expected to significantly enhance the material’s damping capacity by providing a high density of interfaces and defects that effectively dissipate vibrational energy through mechanisms such as interface sliding and dislocation motion [16]. This work thus provides a microstructural foundation for developing high-performance Ti composites for dynamic applications.

## 4. Conclusions

This study systematically investigates the grain refinement and α texture evolution in GNPs/TA15 composites during extrusion. The key findings are as follows: (I) Dynamic recrystallization plays a crucial role in grain refinement, reducing the average grain size from 3.04 μm to 1.30 μm. The process is characterized by dislocation accumulation along GNPs and grain boundaries, leading to the formation of dislocation substructures and subgrains, which is evidenced by the increased fraction of low-angle grain boundaries (LAGBs). As extrusion proceeds, the rotation of these subgrains promotes the formation of recrystallized grains and a significant increase in high-angle grain boundaries (HAGBs), culminating in substantial grain refinement. (II) The combined effects of extrusion force and die constraints induce a unique flow pattern in the Ti matrix near GNPs, where the matrix first reorients towards GNPs and then elongates along the extrusion direction (ED). This deformation behavior facilitates the development of strong [10$\bar{1}$1]//ED and [20$\bar{2}$1]//ED α textures, highlighting the significant influence of GNPs on texture evolution during extrusion.

## Data Availability

The original contributions presented in the study are included in the article, further inquiries can be directed to the corresponding authors.
